# Long-Term Persistance of the Pathophysiologic Response to Severe Burn Injury

**DOI:** 10.1371/journal.pone.0021245

**Published:** 2011-07-18

**Authors:** Marc G. Jeschke, Gerd G. Gauglitz, Gabriela A. Kulp, Celeste C. Finnerty, Felicia N. Williams, Robert Kraft, Oscar E. Suman, Ronald P. Mlcak, David N. Herndon

**Affiliations:** 1 Shriners Hospitals for Children, University of Texas Medical Branch, Galveston, Texas, United States of America; 2 Department of Surgery, University of Texas Medical Branch, Galveston, Texas, United States of America; 3 Sealy Center for Molecular Medicine and the Institute for Translational Sciences, University of Texas Medical Branch, Galveston, Texas, United States of America; 4 Department of Dermatology and Allergology, Ludwig Maximilians University, Munich, Germany; 5 Division of Plastic Surgery, Department of Surgery and Sunnybrook Health Sciences Centre, University of Toronto, Toronto, Canada; Rutgers University, United States of America

## Abstract

**Background:**

Main contributors to adverse outcomes in severely burned pediatric patients are profound and complex metabolic changes in response to the initial injury. It is currently unknown how long these conditions persist beyond the acute phase post-injury. The aim of the present study was to examine the persistence of abnormalities of various clinical parameters commonly utilized to assess the degree hypermetabolic and inflammatory alterations in severely burned children for up to three years post-burn to identify patient specific therapeutic needs and interventions.

**Methodology/Principal Findings:**

Patients: Nine-hundred seventy-seven severely burned pediatric patients with burns over 30% of the total body surface admitted to our institution between 1998 and 2008 were enrolled in this study and compared to a cohort non-burned, non-injured children. Demographics and clinical outcomes, hypermetabolism, body composition, organ function, inflammatory and acute phase responses were determined at admission and subsequent regular intervals for up to 36 months post-burn. Statistical analysis was performed using One-way ANOVA, Student's t-test with Bonferroni correction where appropriate with significance accepted at p<0.05. Resting energy expenditure, body composition, metabolic markers, cardiac and organ function clearly demonstrated that burn caused profound alterations for up to three years post-burn demonstrating marked and prolonged hypermetabolism, p<0.05. Along with increased hypermetabolism, significant elevation of cortisol, catecholamines, cytokines, and acute phase proteins indicate that burn patients are in a hyperinflammatory state for up to three years post-burn p<0.05.

**Conclusions:**

Severe burn injury leads to a much more profound and prolonged hypermetabolic and hyperinflammatory response than previously shown. Given the tremendous adverse events associated with the hypermetabolic and hyperinflamamtory responses, we now identified treatment needs for severely burned patients for a much more prolonged time.

## Introduction

Despite significant advances in therapeutic strategies, e.g., improving resuscitation, enhancing wound coverage, appropriate infection control, and improving treatment of inhalation injury, severe burns remain a devastating injury affecting nearly every organ system and leading to significant morbidity and mortality [1]. Main contributors to adverse outcomes of severely burned patients are profound and complex metabolic changes in response to the initial burn [1], [2]. Burns covering more than 30% total body surface area (TBSA) are associated with stress, inflammatory, and hypermetabolic responses that lead to hyperdynamic circulation, increased body temperature, glycolysis, proteolysis, lipolysis and futile substrate cycling [3]–[5]. These responses are present in all trauma, surgical, or critically ill patients, but the severity and magnitude is unique for burn patients [1]. Marked and sustained increases in catecholamine, glucocorticoid, glucagon, and dopamine secretion are thought to initiate the cascade of events leading to the acute hypermetabolic response with its ensuing catabolic state [3], [6]–[13].

Several studies have indicated that these metabolic phenomena post-burn occur in a timely manner, suggesting two distinct patterns of metabolic regulation following injury [14]. The first phase occurs within the first 48 hours of injury and has classically been called the “ebb phase” [14], [15], characterized by decreases in cardiac output (CO), oxygen consumption, and metabolic rate as well as impaired glucose tolerance associated with its hyperglycemic state. These metabolic variables gradually increase within the first five days post-injury to a plateau phase (called the “flow” phase) associated with hyperdynamic circulation and the above mentioned hypermetabolic state. In the past, general understanding has been that these metabolic alterations resolve with complete wound closure or shortly thereafter [16]. Recent studies, however, are indicative that the hypermetabolic response to burn injury persists beyond wound closure, e.g., we have recently shown that alterations in insulin sensitivity persisted for three years after the initial burn injury [17]. In light of these findings, we hypothesized that a burn injury induces vast hypermetabolic and inflammatory alterations associated with physiologic changes that persist not only for 6 to 12 months post-burn but for three years. To test our hypothesis, we conducted a large prospective study in severely burned pediatric patients and determined hypermetabolic and inflammatory responses over a period of three years post-burn.

## Results

### Demographics

Nine-hundred seventy-seven severely burned children were included in the present study. Characteristics of burn patients are depicted in Table 1. Patients were, on average, 7.5 years of age, 36% were females and 64% were males. Patients suffered from a severe thermal injury involving 50% TBSA burn and a third-degree burn of 37% TBSA. During acute hospitalization, length of hospital/ICU stay was 26 days which results in 0.5 days per percent TBSA burn. Patients were taken back to the OR every 7^th^ day and required on average 4 operations. During acute hospitalization, 32% of the patients suffered from inhalation injury, minor infections occurred in 43% of the patients, sepsis occurred in 10%, multi-organ failure in 16%, and 8% of our patients died (Table 1).

**Table 1:** Patient demographics.

N	977
Gender	
Male n (%)	627 (64)
Female n (%)	350 (36)
Ethnicity	
AA	69
C	141
H	738
Other	29
Age admit (years)	7.5±5.3
Inhalation Injury n (%)	308 (32)
Type of burn	
Flame (n)	721 (74)
Scald (n)	200 (21)
Other (n)	56 (25)
TBSA burn (%)	50±20
TBSA second (%)	21±17
TBSA third (%)	37±26
Burn to admit (days)	13±34
Operations acutely (n)	3.7±3.2
Time between OR's (days)	6±5
LOS ICU (days)	26.2±25.5
LOS/TBSA (days)	0.5±0.4
Died n (%)	75 (8)
Max DENVER2	3.2±1.8
MOF n (%)	155 (16)
Sepsis n (%)	93 (10)
Patients with minor infections n (%)	420 (43)

TBSA  =  total body surface area. Data presented as means ± SD or percentages.

### Hypermetabolism

#### Indirect calorimetry

**Figure 1:**
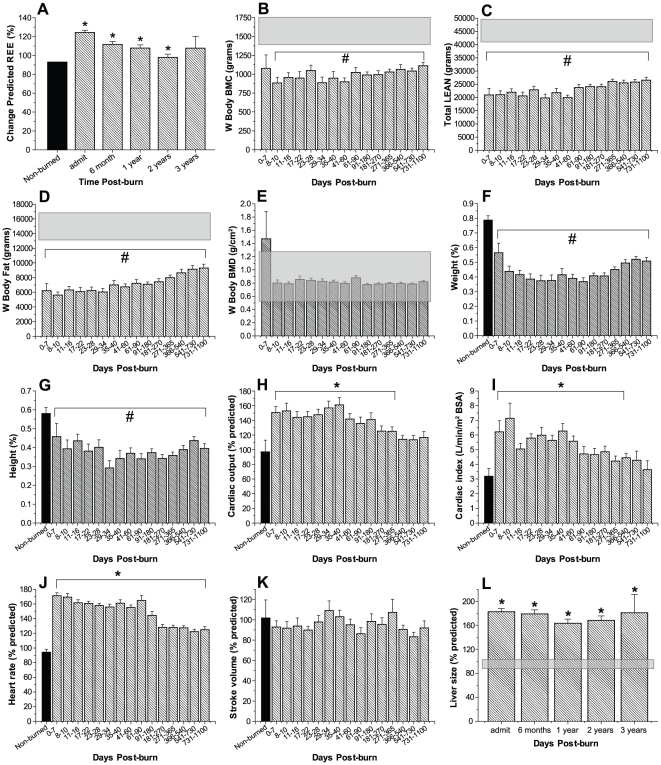
Persistently increased percent predicted REE indicate prolonged hypermetabolism (A). REE % predicted increases upon burn injury and decreased over time but remains significantly elevated up to two years post-injury. Bars represent means; error bars correspond to S.E.M. Asterisks denote statistical difference between burned children vs. normal range, p<0.05. Body composition, weights and heights: Bone mineral content (B), LBM (C), fat (D, E) and BMD (F, G) were measured at admission and subsequent time points. DEXA analysis revealed significantly decreased values throughout the whole time period studied for BMC, LBM, and fat, p<0.05. Cardiac function and liver size post-burn: Percent predicted cardiac output (H) and cardiac index (I) were significantly increased for up to 12 months post-burn. Heart rate (J) was twice that of non-burned children for up to three years post-burn, while predicted stroke volume (K) was normal. Liver size increased by nearly two-fold upon burn injury of predicted liver size and remained elevated for the remaining of the study (L). Bars represent means; error bars correspond to S.E.M. Asterisks denote statistical difference between burned children vs. non-burned children, p<0.05.

Predicted REE increased significantly post-burn and then gradually decreased over time, but remained significantly elevated for two years following burn injury indicating marked hypermetabolism, p<0.05 (Fig. 1A).

#### Body composition, weights and heights

Bone mineral content (Fig. 1B), LBM (Fig. 1C), fat (Fig. 1D, E) and BMD (Fig. 1F, G) were measured at admission and subsequent time points. DEXA analysis revealed significantly decreased values throughout the whole time period studied for BMC, LBM, and fat, p<0.05 (Fig. 1B–E). BMD was not significantly different from normal (Fig. 1F, G).

On admission, the patient population fell within essentially the normal distribution pattern for both height and weight. Thirty-six percent of the patient population fell below the 50^th^ percentile (the mean) for height at admission, while the percentage of burned children that fell below the 50^th^ percentile for height was significantly greater for up to two years post-burn, indicating a profound growth delay in this patient population. Forty-two percent of the patients included this study were below the mean for weight at admission, while the percentage of burned children that fell below the 50^th^ percentile for weight was significantly greater for up to three years post burn, p<0.05. Data demonstrate that it takes approximately 1–2 years for pediatric burn patients to grow again and improve their height and weight percentile.

#### Organ changes

Analysis of CO, CI and HR revealed marked alterations in response to burn. CO increased immediately post-burn and remained significantly elevated over 12 months before gradually decreasing to values of non-burned controls (Fig. 1H). CI was also significantly elevated during the first 12 months post-burn before returning to values of non-burned patients for the remaining of the study (Fig. 1I). While HRs of severely burned pediatric patients vastly increased immediately after burn to values of 173±6% with that of non-burned children, and remained significantly increased over 3 years post-burn, SV of these children was not significantly different from that of normal controls (Fig. 1J, K).

Analysis of liver size determined by ultrasounds demonstrated markedly increased liver size in response to the initial burn trauma for up to three years, p<0.05 (Fig. 1L). Throughout the time period studied, liver size of severely burned children was increased by an average of 75% compared to healthy non-burned children of similar age. Interestingly, we did not detect a decrease in liver size over the three-year study period.

### Inflammatory and acute phase response

#### Urinary catecholamine and cortisol measurements

**Figure 2:**
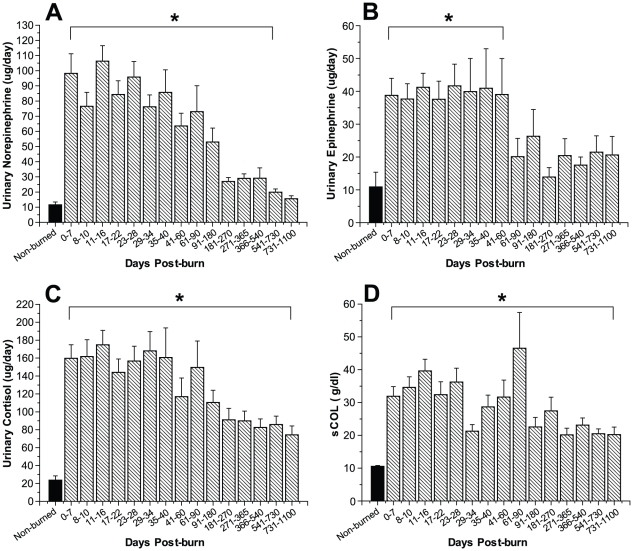
Urinary norepinephrine (A) and epinephrine (B) are significantly increased for two and 18 months post-burn, respectively. Twenty-four hour total urine cortisol (C) and serum cortisol (D) levels increase upon burn injury and remain significantly elevated for up to 36 months. Bars represent means; error bars correspond to S.E.M. Asterisks denote statistical difference between burned children vs. non-burned children, p<0.05.

Urinary norepinephrine, epinephrine, and cortisol increased markedly immediately after burn trauma. Urinary norepinephrine increased 10-fold during the early phase post-burn and remained significantly elevated up to 540 days post-burn when compared with non-burned control patients, p<0.05 (Fig. 2A). Urinary epinephrine levels significantly increased 4- to 5-fold post-burn and remained elevated for 60 days post-burn, p<0.05 (Fig. 2B). Total urine cortisol levels initially increased 8- to 10-fold and levels remained significantly increased 3 years post-burn, p<0.05 (Fig. 2C). Serum cortisol measurements displayed similar characteristics and levels were significantly increased up to 3 years post-burn, p<0.05 (Fig. 2D).

#### Serum cytokines

**Figure 3:**
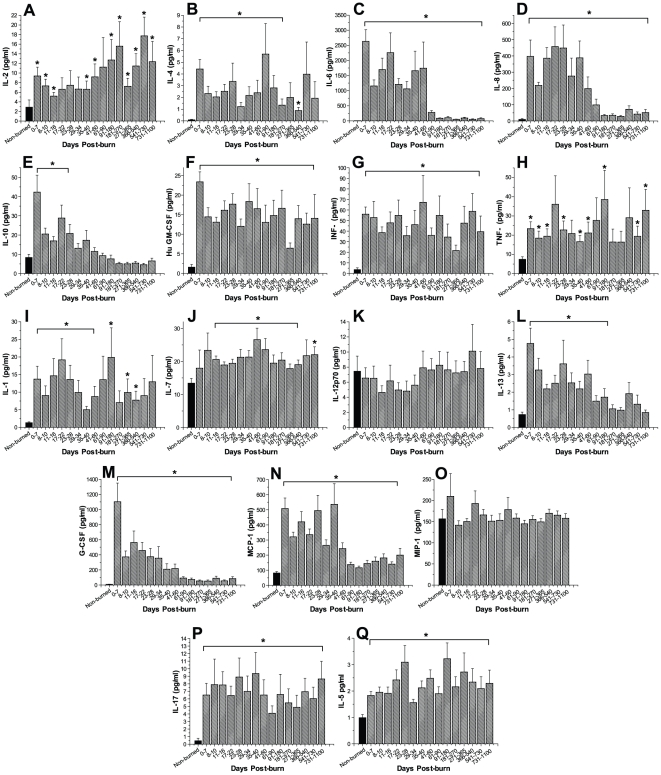
Severe burn leads to markedly increased inflammatory response. Fourteen cytokines measured within this study were significantly altered in response to burn injury. Particularly serum IL-6, IL-8, G-CSF and MCP-1 revealed dramatic increases. IL-12p70 and MIP-1β were not significantly altered in response to burn trauma when compared to controls. Histograms depict serum concentrations of the respective cytokine at steady state levels. Bars represent means; error bars correspond to S.E.M. Asterisks denote statistical difference between burned children vs. non-burned children, p<0.05.

We found that almost all cytokines measured within this study were significantly altered in response to burn injury (Fig. 3A–Q). Dramatic changes were observed for serum IL-6, IL-8, G-CSF and MCP-1 (Fig. 3C, D, M, N). These cytokines demonstrated an up to 2,000-fold increase immediately upon burn trauma and remained significantly elevated throughout the time period studied when compared with the concentrations detected in non-burned controls, p<0.05. GM-CSF, INF-γ, TNF-α, IL-1β, IL-2, IL-5, IL-7, IL-10, and IL-17 significantly increased by 2- to 20-fold in response to burn injury and revealed relatively constant, but significantly increased levels for most of the three-year period post-burn compared to non-burned patients (p<0.05). IL-12p70 and MIP-1β were not significantly altered in response to burn trauma when compared to controls.

#### Serum proteins

**Figure 4:**
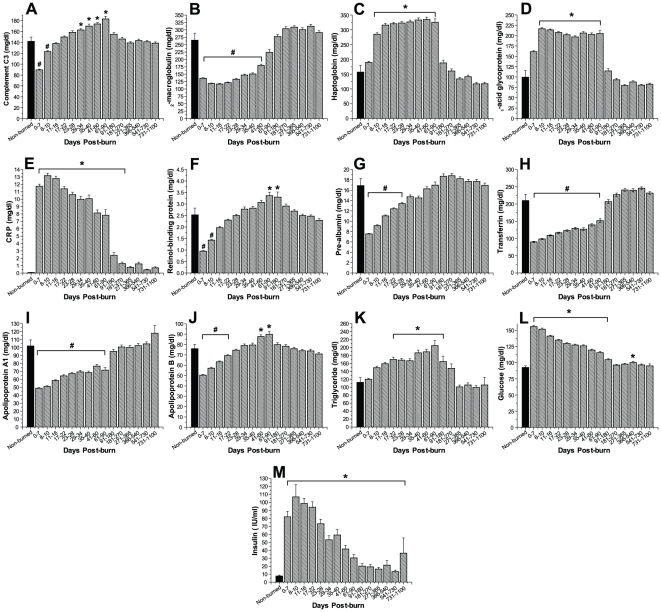
Serum proteins. Acute phase proteins and constitutive proteins are significantly altered for up to 18 months post-burn. Serum complement C3 (A), α2-macroglobulin (B), haptoglobin (C), α1-acidglycoprotein (D), and CRP (E) were significantly increased for up to nine months post-burn. Serum constitutive hepatic proteins retinol binding protein (F), pre-albumin (G), transferrin (H) markedly decreased immediately post-burn and remained diminished for up to six months post-burn. Serum apolipoprotein A1 (I) and apolipoprotein B (J) were markedly decreased for 18 and one month, respectively. Serum triglycerides (K) demonstrated significantly increased levels for nine months post-burn. Burn trauma leads to hyperglycemia and elevated fasting serum insulin concentrations, indicating insulin resistance. Histograms depict fasting serum concentrations of (L) glucose and (M) insulin. Bars represent means; error bars correspond to S.E.M. Asterisks denote statistical difference between burned children vs. non-burned children, p<0.05.

Serum acute phase proteins were significantly altered upon burn injury (Fig. 4A–L). Serum complement C3 concentrations initially demonstrated significantly diminished levels compared to those of non-burned controls, before peaking at 29 to 90 days post-burn with significantly elevated levels and then rapidly decreased to basal levels for the remaining of the study period, p<0.05 (Fig. 4A). Serum α_2_-macroglobulin concentrations displayed significantly decreased values for up to 60 days post-burn before gradually increasing to levels of non-burned controls, p<0.05 (Fig. 4B). Serum haptoglobin, α_1_-acidglycoprotein, and CRP demonstrated a 2- to 12-fold increase immediately upon burn injury and remained significantly elevated for up to 90 and 270 days post-burn, respectively, compared to non-burned controls, p<0.05 (Fig. 4C–E). Serum constitutive hepatic proteins retinol-binding protein, pre-albumin and transferrin markedly decreased by 2-fold immediately post-burn and remained significantly decreased for up to 90 days post-burn, p<0.05 (Fig. 4F–H). Serum apolipoprotein A1 significantly decreased post-burn and remained significantly diminished for a period of 90 days, p<0.05 (Fig. 4I). Apolipoprotein B demonstrated a diminutive initial decrease then steadily increased to significantly elevated levels between 41 and 90 days before gradually decreasing to basal levels of non-burned patients, p<0.05 (Fig. 4J). Serum triglycerides gradually increased upon burn injury and demonstrated significantly elevated levels between 17 to 180 days post trauma (Fig. 4K).

Serum glucose significantly increased immediately upon burn injury to levels of 156±2 mg/dl and remained significantly elevated for a period of 180 days before gradually decreasing to levels within the normal physiologic range, p<0.05 (Fig. 4L). Serum insulin levels also rapidly increased to significant levels in response to burn, before subsequently decreasing but remaining significantly elevated for the whole time period studied when compared with the serum concentrations detected in non-burned controls, p<0.05 (Fig. 4M).

**Figure 5:**
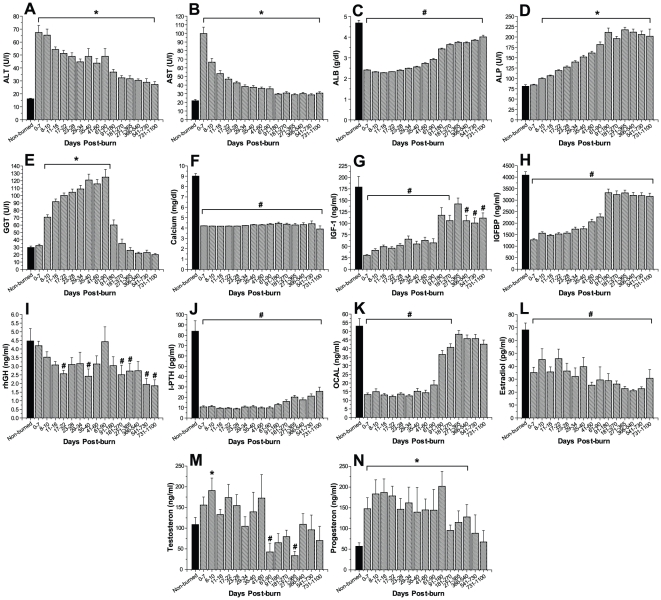
Hepatic enzymes and proteins. Histograms depict serum concentrations of Alanin-Aminotransferase (ALT) (A), aspartat-aminotransferase (AST) (B), albumin (ALB) (C), alkaline phosphatase (ALP) (D), glutamyl transpeptidase (GGT) (E), and serum calcium concentrations (F). Serum IGF-I (G), IGFBP-3 (H), GH (I), iPTH (J), osteocalcin (K), EST (L) were significantly decreased in response to thermal injury and remained diminished for up to three years post-burn. Analysis of serum testosterone (M) and progesterone (N) revealed only moderate increases throughout the first two months post-burn. Bars represent means; error bars correspond to S.E.M. Asterisks denote statistical difference between burned children vs. non-burned children, p<0.05.

Serum concentrations of Alanin-Aminotransferase (ALT) and aspartat-aminotransferase (AST) significantly increased immediately upon burn trauma and remained significantly elevated for the remaining of the study period, p<0.05 (Fig. 5A, B). Serum Albumin (ALB) concentrations demonstrated significantly decreased levels for the entire three-year period compared to non-burned controls, p<0.05 (Fig. 5C). Both, alkaline phosphatase (ALP) and gamma glutamyl transpeptidase (GGT) were significantly altered in response to burn. While ALP displayed significantly elevated levels starting 8 days post-burn injury, which remained significantly elevated for the remaining of the study, serum concentrations of GGT raised to significant values beginning eight days post-burn before rapidly decreasing to normal concentrations beginning 90 days post-trauma, p<0.05 (Fig. 5D, E). Serum calcium concentrations, however, displayed significantly decreased levels for the entire three-year period, p<0.05 (Fig. 5F).

#### Serum hormones

All serum hormones measured within this study demonstrated significant alterations in response to burn trauma. Serum levels of both, insulin growth factor (IGF)-I and insulin-like growth factor binding protein-3 (IGF BP-3), decreased significantly immediately post-burn and remained diminished for most of the remaining study period, compared to levels of non-burned controls, p<0.05 (Fig. 5G, H). Human growth hormone (hGH) gradually declined in response to burn injury over the time period studied and demonstrated significantly decreased values for several of the time points within the three-year period when compared to controls, p<0.05 (Fig. 5I). Serum parathormone (iPTH) decreased by 8-fold immediately post-burn and remained significantly decreased for three years post-burn, p<0.05 (Fig. 5J). Serum levels of osteocalcin also displayed significantly decreased values for a time period of 270 days before rapidly increasing to levels of non-burned controls, p<0.05 (Fig. 5K). Serum concentrations of estrogen (EST), testosterone (TEST) and progesterone (PROG) displayed diverse patterns post-burn injury. While serum estrogen decreased immediately post-burn and remained significantly decreased for the entire time period studied, p<0.05 (Fig. 5L), serum testosterone gradually increased upon burn trauma with significant levels at 8 to 10 days post-burn, respectively, before gradually decreasing to diminished levels beginning 60 days post-burn, p<0.05 (Fig. 5M). Serum progesterone concentrations displayed significantly elevated levels for the first two years post-burn before gradually decreasing to values of non-burned controls, p<0.05 (Fig. 5N).

## Discussion

The importance of this study is that it clearly demonstrated that burn induced metabolic and inflammatory changes persisted for 3 years after the injury. The relevance of post-burn hypermetabolism and inflammation is that they induce insulin resistance for 3 years [17], 50- to 100-fold increase in fracture risk [1], 200% increase in liver-size [18], [19], growth and development retardation for 2–3 years [3], increased cardiac work and develop cardiac dysfunction [20], impaired strength [3], [20], muscle function, hormonal abnormalities [17], [18], increased risk for infections and sepsis [3], [20]. All the aforementioned can lead to morbidity and mortality of the patient. We now showed that this risk to die is not over when the patient is 95% healed; it persists for up to 3 years post-burn.

Even though the metabolic alterations after severe burn injury are similar to any major trauma, severe burns are characterized by a hypermetabolic response that is more severe and sustained than any other form of trauma [16]. Several studies have extensively delineated the complexity of the acute post-burn pathophysiologic response [14], [15], [18]; however, it is currently unknown how long these metabolic phenomena persist beyond the first 12 months after the initial event [6], [13], [21], [22]. Marked and sustained increases in catecholamine, glucocorticoid, and glucagon secretion are thought to initiate the cascade of events leading to the acute hypermetabolic response with its ensuing catabolic state [3], [6]–[13]. Contrary to past understanding that these metabolic mediators resolve soon after complete wound closure [16], we could demonstrate catecholamines and stress hormones such as cortisol were elevated for up to 36 months post-burn accompanied by significant increases in REE indicative of vast hypermetabolism. Resting metabolic rates in burn patients have been shown to increase in a curvilinear fashion, ranging from near normal for burns less than 10% TBSA to twice that of normal in burns more than 40% TBSA. In patients with burn injuries greater than 40% TBSA, resting metabolic rate at thermally neutral temperature (33°C) reaches up to 180% of the basal rate during acute admission, 150% at full healing of the burn wound, 140% at six months after the injury, 120% at nine months after injury, and 110% after 12 months [3]. In this study, we could demonstrate that even three years after the initial trauma REE is still above normal, indicating a persistent hypermetabolic response. The exact cause of this complex response, however, is still poorly understood. IL-1 and -6, platelet-activating factor, TNF, endotoxin, neutrophil-adherence complexes, reactive oxygen species, nitric oxide and coagulation as well as complement cascades, all have been implicated in regulating this response to burn injury [23]. Here, we found marked alterations for 14 cytokines in response to burn injury. Particularly, serum IL-6, IL-8, G-CSF and MCP-1 displayed dramatic changes. These cytokines demonstrated an up to 2000-fold increase immediately post-burn and remained significantly elevated throughout the time period studied. Cytokines are the primary mediators of this inflammatory reaction to injury [24]. They constitute a group of proteins with autocrine and endocrine activities that provide communication among different types of cells, including those that mediate immune functions, angiogenesis, cell proliferation and apoptosis [24]. Inflammatory cytokines such as TNF, IL-6 and MCP-1 have been also shown to inhibit insulin action through modification of signaling properties of insulin receptor substrates, contributing to liver and skeletal muscle insulin resistance [25]–[27].

Persistently increased glucose and insulin levels as shown in this study are of serious clinical concern since hyperglycemia has been frequently linked to impaired wound healing [28], increased skin graft loss [29], increased muscle protein catabolism [30], increased incidence of infections [31], [32] and mortality [2], [31]–[35]. Maintaining blood glucose at levels below 110 mg/dl using intensive insulin therapy has been shown to reduce mortality and morbidity in critically ill patients [36]; however, associated hypoglycemic events have led to the investigation of alternative strategies, including the use of metformin [37] and the PPAR-γ agonist fenofibrate [38]. Other underlying factors for the observed elevated glucose and insulin levels may include the above mentioned prolonged increases in endogenous stress hormones, which have been causally associated with injury-induced insulin resistance [8]–[13]. Also, decreases in muscle mass, both during the acute and recovery phases following injury, may significantly contribute to this persistent insulin resistance, since skeletal muscle has been shown to be responsible for 70–80% of whole-body insulin-stimulated glucose uptake [39]. In contrast to starvation, in which lipolysis and ketosis provide energy and protect muscle reserves, burn injury considerably reduces the ability of the body to utilize fat as an energy source. Skeletal muscle is thus the major source of fuel in the burned patient, which leads to marked wasting of LBM within days after injury [1], [40], as shown in our burned patients. Increased protein turnover, degradation, and negative nitrogen balance are common characteristics of severe burn trauma [41]. As a consequence, structure and function of essential organs such as skeletal muscle, skin, immune system, and cellular membrane transport functions may be compromised [42], [43]. In 1998, Chang and colleagues [44] defined that a 10% loss of LBM may lead to impaired immune function, a 20% loss of LBM to impaired wound healing with an associated 30% mortality, a 30% loss of LBM to pneumonia and pressure sores with an associated 50% mortality, and a 40% loss of LBM may ultimately result in death in 100% of cases.

Other significant observations in this large prospective trial include substantially affected expression of acute phase proteins. Particularly haptoglobin, α_1_-acidglycoprotein, and CRP demonstrated significant increases for up to nine months post-burn. Serum constitutive hepatic proteins, in contrast, such as retinol-binding protein, pre-albumin and transferrin, were found to be significantly decreased for up to six months post-injury. This decrease could be due to decreased production, increased consumption or increased loss due to capillary leakage. These proteins represent commonly utilized markers for general homeostasis indicating the severity and intensity of the prolonged post-burn dysbalance [18]. Determinations of serum triglycerides revealed significant increases for nine months post-trauma, a finding which may help explain the commonly observed fatty infiltration of liver and other organs of burn victims. A recently demonstrated association between hepatomegaly with fatty infiltration and increased incidence of sepsis and mortality supports the importance of this observation [45]. After thermal injury, a variable degree of liver injury is present, and it is usually related to the severity of the thermal injury. Fatty changes, a very common finding, are per se reversible and their significance depends on the cause and severity of accumulation [45]–[50]. In this study, analysis of liver ultrasounds demonstrated markedly increased liver size in response to the initial burn trauma for up to three years. Other hepatic parameters utilized to determine liver function, including ALT and AST, were significantly altered for the entire study period, also indicating that liver damage is present for a prolonged period post-trauma. As described previously by our group, serum apolipoprotein A1 significantly decreased upon burn trauma and remained significantly diminished for three years, apolipoprotein B, in contrast, only demonstrated diminutive initial decreases before returning to normal values [18]. The exact role of these two proteins in this context, however, remains to be determined.

As recently demonstrated by Jeschke et al. [18] in the acute phase post-burn, several hormonal axes are affected by burn trauma. Overall, critical illness is characterized by marked alterations in the hypothalamic-anterior-pituitary-peripheral-hormone axes, the severity of which is associated with a high risk of morbidity and mortality [51]. Within this study, we also found prolonged alterations in GH-IGF-I-IGFBP-3-axis, PTH-Osteocalcin axis, and sex hormones (testosterone, β-estradiol, progesterone). Particularly, serum levels of IGF-I and IGF BP-3 demonstrated a substantial decrease for up to three years post-burn while measured levels of hGH were rather moderately decreased for the whole time period studied. Beneficial effects of recombinant human growth hormone (*rhGH*) in trauma patients have been demonstrated in various settings. Besides enhancing immune function [52], [53], wound healing [54], and decreasing the overall hypermetabolic response after major surgery, trauma, sepsis or a thermal injury [55]–[57], *rhGH* stimulates protein synthesis and attenuates the nitrogen loss after injury and improves clinical outcomes [58]. Also, *rhGH* modulates the hepatic acute phase response by increasing constitutive hepatic proteins, decreasing acute phase proteins, modulating cytokine expression, and increasing IGF-I concentrations [59], [60]. However, ever since *rhGH* administration in trauma patients has been shown to increase mortality in a prospective, randomized, double-blind study by Takala and colleagues [61], the use of *rhGH* has been restricted. Administration of IGF-I may thus represent a promising therapeutic alternative, since recent studies could demonstrate that IGF-I, in combination with its principle binding protein, improved muscle protein synthesis, hepatic acute phase and inflammatory response, and the immune system [62]–[64].

Determination of serum osteocalcin and parathyroid hormone levels also demonstrated significant decreases for nine and up to 36 months, respectively, associated with profound decreases in BMC and BMD. As shown by Klein and others [10], [65]–[70] using labeled tetracycline in order to determine bone turnover rates, severely burned patients are lacking overall bone formation and synthesis. Besides administration of pamidronate, which was recently shown to improve bone metabolism during the acute phase and long-term phase post-burn [69], sex hormone substitution may represent a potential therapeutic approach. Particularly, since estrogen levels were diminished for the whole time period studied and estrogen substitution has been shown to improve bone mineralization and metabolism [71].

Analysis of various cardiac parameters showed marked alterations in response to burn. Percent of normal CO as well as CI were significantly elevated during the first 180 days post-burn, accompanied by a massive tachycardia with 120–180% predicted HR for the whole time period studied. Elevated levels of plasma catecholamines instigate the cardiac stress post-burn. Plasma catecholamine levels are elevated up to nine months post-burn, but the derangements in cardiac physiology last up to three-years after the initial trauma. Elevated catecholamines increase myocardial oxygen delivery, myocardial oxygen consumption and cause focal degeneration of the myocardium and hypertrophy. In excess, they cause cardiac deficiency, local myocardial hypoxia, and cardiac death [72]. Thus, prolonged exposure to catecholamine levels 10-fold higher than normal is cause for clinical concern. However, the long-term ramifications of the cardiac stress seen post-burn is still unknown. Initially, it was thought that these derangements would subside shortly after the acute hospitalization or the initial resuscitation. More recent research has shown that these responses may last 9–12 months after the initial insult [16], [73], [74]. Here, we demonstrated a significant increase in cardiac work up to three years after the initial injury. This may be a profound detriment to our pediatric burn patients by increasing morbidity and leading to long-term cardiac exhaustion, or cardiovascular complications in the future. These findings support the use of an anti-catabolic agent to attenuate the effects of increased catecholamines, beta-adrenergic regulation, and the need for cardiovascular protection.

Therapeutic advancements in the acute phase post-burn, such as early excision and closure of the burn wound, more appropriate infection control and anti-catabolic therapeutic intervention, including beta-adrenergic blockade with propranolol, growth hormone, insulin-like growth factor, oxandrolone, testosterone, and insulin have substantially contributed to significant improvement of morbidity and mortality rates in burn patients during acute hospitalization. However, based on this study, we suggest that a severe burn is not an acute illness but rather a chronic health problem. We thus believe that burn patients should be carefully monitored for at least 3–4 years in order to reverse these complex metabolic alterations post-burn.

## Materials and Methods

### Patients

All thermally injured pediatric patients with burns covering more than 30% of their TBSA were admitted to our institution between 1998 and 2008, required at least one surgical intervention, and consented to the University of Texas Medical Branch Institutional Review Board-approved experimental protocol were included in this study. Patient, parent or legal guardian provided with written consent for participation in the study.

### Admission Data

On admission, the extent and degree of burn was assessed and recorded on a standard Lund and Browder chart by the attending burns surgeon present. Information also recorded at the time of admission included burn related (date and mechanism) as well as demographic data (age and gender). All patients were treated in our pediatric burns intensive care unit according to standardized protocols.

Patients were resuscitated if needed according to the Galveston formula with 5000 cc/m^2^ TBSA burned +2000 cc/m^2^ TBSA lactated Ringer's solution given in increments over the first 24 hours. Within 24 hours of admission, all patients underwent total burn wound excision and the wounds covered with available autograft skin and any remaining open areas were covered with homograft. After the first operative procedure, it took 5–10 days until the donor site was healed and patients were taken back to the operation theater. This procedure was repeated until all open wound areas were covered with autologous skin material.

All patients underwent the same nutritional treatment according to a standardized protocol. The intake is calculated as 1500 kcal/m^2^ body surface +1500 kcal/m^2^ area burn or we assessed the need by measuring the resting energy expenditure (REE), multiplied by 1.4 with weekly adjustments as previously published [6], [22].

### Patient demographics

Patient demographics (age, date of burn and admission, gender, burn size and depth of burn) and concomitant injuries such as inhalation injury, sepsis, morbidity, and mortality were recorded. Minor infection was defined as a positive tissue culture with more than 10^5^ colony forming units per gram tissue. Sepsis was defined as a positive blood culture or pathologic tissue culture identifying the pathogen during hospitalization or at autopsy, in combination with at least 3 of the following: leucocytosis or leucopenia (>12,000 or <4,000), hyperthermia or hypothermia (>38.5 or <36.5°C), tachycardia (>150 BPM in children), refractory hypotension (systolic BP <90 mmHg), thrombocytopenia (platelets <50,000/mm^3^), hyperglycemia (serum glucose >240 mg/dl), and enteral feeding intolerance (residuals >200 cc/hr or diarrhea >1 L/day) as previously published [3], [75]. Time between operations was determined as a measurement for wound healing/re-epithelization. As demonstrated previously, we believe that time between operations may be indicative when donor sites were healed and thus allow determination of wound healing.

### Time points

Results obtained during the three-year period were divided into fifteen different time phases: 0 to7, 8 to 10, 11 to 16, 17 to 22, 23 to 28, 29 to 34, 35 to 40, 41 to 60, 61 to 90, 91 to 180, 181 to 270, 271 to 365, 366 to 540, 541 to 730 and 731 to 1,100 days post-burn. Data presented include a number of 31 to 307 different measurements at each time point. If any patient had more than one measurement performed during the respective time period, results were averaged to give a single mean result for each patient at each time period. One-hundred seven non-burned children, who consented for research studies and required blood and/or 24-hour urine collections, were used as normal cohort.

### Hypermetabolism

#### Indirect calorimetry

As part of our routine clinical practice, all patients underwent REE measurements weekly during acute hospitalization and during admissions for reconstructive operations for up to three years post-burn. REE was measured using a Sensor-Medics Vmax 29 metabolic cart (Yorba Linda, CA, USA) as previously published [6]. REE was calculated from the oxygen consumption and carbon dioxide production by equations described before [6]. For statistical comparison, measured energy expenditure was expressed as the percentage of the basal metabolic rate predicted compared with predicted norms based upon the Harris-Benedict equation and to body mass index (BMI) [6].

#### Growth measurements and body composition

Heights and weights were measured during acute hospitalization subsequent stays for reconstructive purposes and were plotted on standard growth charts [76] to obtain the individual height and weight percentiles for age and gender. Percentages of the population plotted within each percentile ranking were then calculated. Total lean body mass (LBM), fat, bone mineral density (BMD), and bone mineral content (BMC) were measured by dual energy x-ray absorptiometry (DEXA). A hologic model QDR-4500W DEXA (Hologic Inc, Waltham, MA) was used to determine body composition as previously published [7], [21], [77], [78].

#### Organ changes

M-Mode echocardiograms were completed as follows: at the time of the study, none of the patients presented with or previously suffered from other concomitant diseases affecting cardiac function, such as diabetes mellitus, coronary artery disease, long-standing hypertension, or hyperthyroidism. Study variables included: resting CO, cardiac index (CI), stroke volume (SV), resting heart rate (HR) and left ventricular ejection fraction (LVEF). SV and CO were adjusted for body surface area and expressed as indexes. All cardiac ultrasound measurements were made with the Sonosite Titan echocardiogram with a 3.5 MHz transducer. Recordings were performed with the subjects in a supine position and breathing freely. M-Mode tracings were obtained at the level of the tips of the mitral leaflets in the parasternal, long axis position and measurements were performed according to the American Society of Echocardiography recommendations. Left ventricular volumes determined at end diastole and end systole were used to calculate EF, SV, CO and CI. Three measurements were performed and averaged for data analysis [21], [78]. Liver size was determined by ultrasound as previously published [18].

### Inflammatory and acute phase response

#### Urinary catecholamine and cortisol measurements

Twenty-four-hour urine collections were taken regularly throughout acute hospital stay and during admissions for reconstructive operations and rehabilitation services. These samples were collected and chilled by the bedside prior to transport to our clinical lab for processing using HPLC techniques as previously published [17]. Extraction of the catecholamines from acidified urine samples were performed using a Bio-Rad kit (Bio-Rad, Hercules, CA), according to manufacturer's instructions.

#### Serum cytokine, protein and hormone measurements

Blood was collected from the burn patients at the time of admission, pre-operatively, and every Monday and Thursday at 6:00 AM, as well as during subsequent stays for surgical and rehabilitation services for serum cytokine and hormone analysis per hospital protocol. Blood was drawn in a serum-separator collection tube and centrifuged for 10 minutes at 1320 rpm; the serum was removed and stored at −70°C until assayed.

Serum hormones were determined using HPLC and ELISA techniques. The Bio-Plex Human Cytokine 17-Plex panel was used with the Bio-Plex Suspension Array System (Bio-Rad, Hercules, CA) to profile expression of Interleukin (IL)-1β, IL-2, IL-4, IL-5, IL-6, IL-7, IL-8, IL-10, IL- 12p70, IL-13, IL-17, granulocyte colony-stimulating factor (GCSF), granulocyte-macrophage colony-stimulating factor (GM-CSF), interferon-gamma (IFN-γ), monocyte chemoattractant protein-1 (MCP-1), macrophage inflammatory protein-1-beta (MIP-1β), and tumor necrosis factor (TNF). The assay was performed according to the manufacturer's instructions. Acute phase and constitutive protein levels were measured using BN II Plasma Protein Analyzer (Dade Behring/Siemens Healthcare, Deerfield, IL) by a nephelometric technique, according to manufacturer's instructions.

### Ethics and statistics

The study was reviewed and approved by the Institutional Review Board of the University Texas Medical Branch, Galveston, Texas. Prior to the study, each subject, parent or child's legal guardian signed a written informed consent form. Analysis of variance (ANOVA) with post hoc Bonferroni correction, paired and unpaired Student's t-test, Chi-square analysis, and Mann-Whitney tests were used where appropriate. Data are expressed as means±SD or SEM, where appropriate. Significance was accepted at p<0.05.

### Study oversight

This study was registered at www.clinicaltrials.gov: #NCT00239668 and #NCT00673309. A steering committee consisting of academic investigators designed the study and monitored its conduct. Data were collected by the investigators and analyzed by scientists. All the authors had access to the data, participated in the data analysis and interpretation, and wrote the manuscript. All authors vouch for the accuracy and completeness of the data and the statistical analysis. All authors participated in the writing of the manuscript and approved the final manuscript before submitting it for publication.
